# Cellular, bone-like tissue in the bucklers and thorns of the thornback ray *Raja clavata* (Batoidea, Chondrichthyes)

**DOI:** 10.1098/rspb.2025.0489

**Published:** 2025-09-03

**Authors:** Aaron R. H. LeBlanc, Moya Meredith Smith, Melanie Debiais-Thibaud, Esther Manzanares, Mason Dean, Charlie Underwood, Zerina Johanson

**Affiliations:** ^1^ Faculty of Dentistry, Oral & Craniofacial Sciences, King's College London, London, UK; ^2^ Natural History Museum, London, UK; ^3^ Institut des Sciences de L'evolution, Montpellier, Languedoc-Roussillon, France; ^4^ Department of Infections Disease and Public Health, City University of Hong Kong, Kowloon Tong, Hong Kong; ^5^ Department of Earth and Planetary Sciences, Birkbeck College, London, UK; ^6^ Department of Palaeontology, Natural History Museum, London, UK

**Keywords:** Chondrichthyes, bone, Batoidea, *Raja clavata*, skin denticles, bucklers, thorns, placoid scales

## Abstract

Chondrichthyans (cartilaginous fishes) have lost the cellular bone characteristic of other jawed vertebrate skeletons. However, we identify cellular bone-like tissue in modified scales with enlarged bases, called ‘bucklers’ and ‘thorns’, which are distinctive for one group of extant batoids (rays). As placoid scales, they possess spines of orthodentine and osteodentine, but a unique basal structure. This consists of a cell-rich material, previously misidentified as an acellular tissue. Newly formed basal tissue grows appositionally and episodically from a cell-rich periosteum-like layer and closely resembles cellular bone, with entombed cells situated between bundles of attachment fibres anchoring the scale to the underlying dermal tissue and the ‘periosteum’ to the scale surface. In histologically more mature tissue, the cell spaces and attachment fibres are remodelled, forming enlarged, elongated spaces. The result is a unique mineralized tissue in these rays, initially sharing similarities with cellular bone, but with a mature state where cell spaces are modified throughout the base, by proposed remodelling of the matrix. Our findings of cellular bone forming the attachment tissues in ray scales demonstrate the chondrichthyan capacity to deposit bone-like tissues within the odontode module, contrary to previous understandings of hard tissue evolution in vertebrates.

## 1. Introduction

Among the chondrichthyans (cartilaginous fishes), sharks (Elasmobranchii) are well-known for their placoid scales or skin denticles. In most sharks, these are small, normally closely packed scales covering the outer skin, with different morphologies depending on location and function [1–5]. Placoid scales are, however, less studied in the other major group of elasmobranch chondrichthyans, the Batoidea (rays, skates), where denticles may form a complete covering, as in sharks, or comprise larger denticles located at specific points along the body with either smaller, shark-like denticles or naked skin between them. As a result of this diversity, a unique terminology has been applied to ray denticles, including small ones more comparable to those in sharks (‘prickles’) and those with larger bases and recumbent spines (‘thorns’; [6–8]). Thorns are modified denticles normally located at specific points on the head (e.g. around the orbits), pectoral fins and in rows along the body [7]. Additionally, some of these thorns possess highly enlarged bases known as bucklers [8]. Their bases are so enlarged that they expand not only ventrally but also dorsally to impinge upon the central tooth-like spines. The presence and distribution of bucklers is sometimes inconsistent between species of the same genus, and even individuals of the same species (C. Underwood, personal observation, 2024), making it difficult to ascribe a function to these enlarged scales.

Reif [9] investigated the histology of bucklers in the ray *Raja*, interpreting the composition of the expanded base to include an unusual acellular, microcancellous bone with incremental growth lines. This interpretation of bone in the bucklers is intriguing, considering that bone has reportedly been lost during the evolution of the chondrichthyans [10,11]. For this reason, we investigated the histology and formation of bucklers and thorns in extant *Raja clavata*, combining computed tomography (CT) scanning, ground and paraffin section histology, and elemental imaging to identify the major tissues and their growth patterns.

Our aim was to use a multimodal approach to reveal the detailed hard and soft tissue properties, as well as potential cellular content of the buckler and thorn tissues. We hypothesized that ground and paraffin sections would reveal whether the buckler and thorn tissues were unique among vertebrates or were similar to true bone. Previous authors have investigated whether bone or bone-like tissues are found in extant chondrichthyans, however, considerable debate remains [12–16]. If the bucklers do indeed contain bone, these structures would demonstrate that, despite losing bone early in their evolutionary history, chondrichthyans can still produce a diversity of mineralized tissues within the odontode, the fundamental developmental module in the evolution of teeth and related vertebrate structures.

## 2. Material and methods

### (a) Specimens

**Figure 1.**
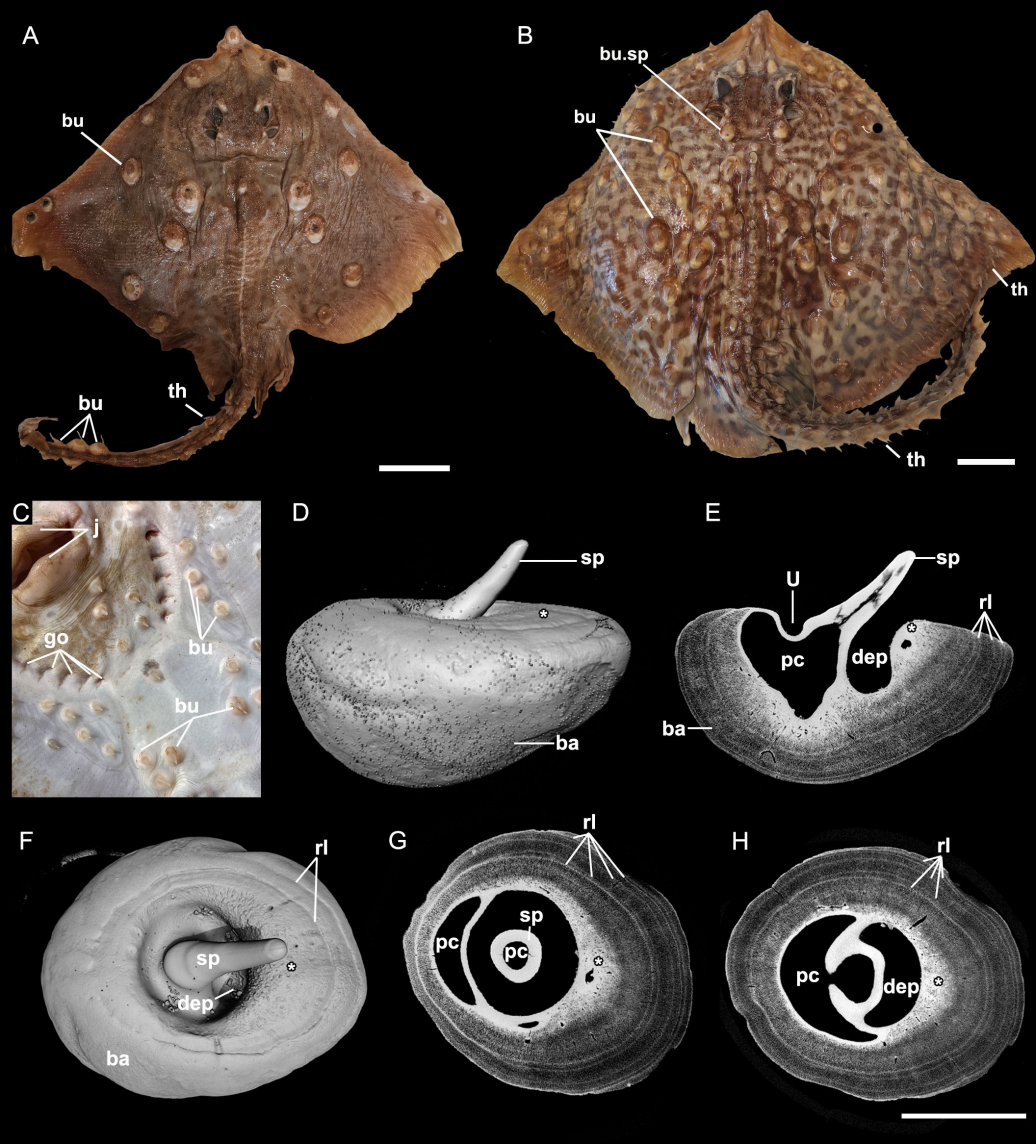
Distribution of thorns and bucklers in extant female *Raja clavata* (Rajidae, Chondrichthyes) and the gross external and internal anatomy of a buckler. A, BMNH 64.4.26:87-8, smaller individual dominated by symmetrically positioned bucklers; B, BMNH 85.11.3.4, larger, putatively older individual with more bucklers present, more thorns have been added (on the tail, fin edges). C, Ventral view of a freshly caught *R. clavat*a showing numerous bucklers around the jaws, gill openings and farther posteriorly (specimen was released after being photographed). D, Lateral view of a CT scanned buckler (BMNH 2017.5.12.9, NHM Life Sciences collection) showing the expanded base. E, Virtual longitudinal section. F, Dorsal view of the buckler. G, Virtual horizontal section through the buckler near the dorsal surface of the basal tissue. H, Virtual horizontal section farther ventrally than in G. Scale bars in A, B = 5 cm, scale bars in D–H = 0.5 cm. ba, buckler base; bu, buckler; bu.sp, buckler near spiracle; dep, posterior depression of buckler spine; go, gill opening; j, jaws; pc, pulp cavity; rl, rest line; sp, spine of buckler spine; th, thorn; U, ‘U’-shaped bend along anterior margin of the spine. White asterisk indicates the position of posterior overgrowth of tissue impinging upon the spine.

**Figure 2.**
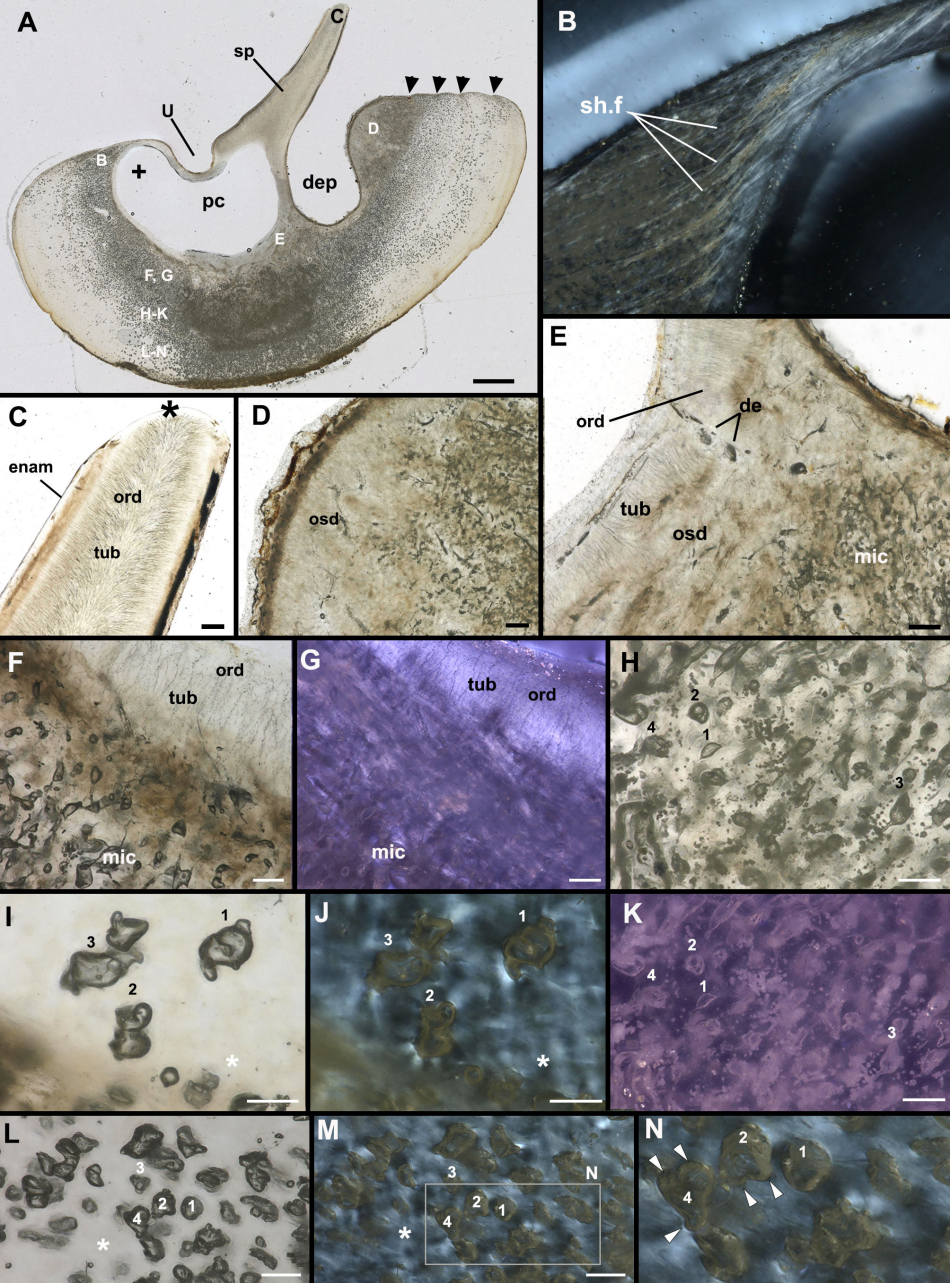
Hard tissue histology of the major tissue types in a buckler of *Raja clavata* (Rajidae, Chondrichthyes, unregistered specimen, NHM Life Sciences collection). A–N, sections through buckler shown in figure 1. A, C–F, H, I, L, plane-polarized light; B, G, J, K, M, N, cross-polarized light. A, Longitudinal serial section, letters B–N indicate regions in close-up on the remainder of the plate, black arrow indicates basal layer shown in more detail in figure 5. Black arrowheads indicate growth lines; B, point where the spine meets the buckler base, dominated by Sharpey’s fibres; C, tip of the buckler spine, asterisk indicates wear at the tip; D, posterior margin of the spine (equivalent to region marked by asterisks in figure 1 D–F); E, base of the spine with ortho- and osteodentine, buckler base below with microcancellous tissue; F, G, base of the spine with orthodentine and microcancellous tissue below; H, K, microcancellous tissue with denser spaces; I, J, microcancellous tissue with less dense spaces, intrinsic fibres (asterisk) apparent in cross-polarized light (J); L–N, microcancellous tissue with dense intrinsic fibres distributed throughout; N, close-up of region in M, to show scalloped surfaces indicative of osteoclast-like activity and resorption (arrowheads). de, denteon; enam, enameloid; mic, microcancellous tissue; ord, orthodentine; osd, osteodentine; sh.f, Sharpey’s fibres; tub, dentine tubules; numbers 1−4 indicate equivalent microcancellous tissue spaces in F, G, H, K, I, J, L–N. Scale bars: A = 1000 µm, B–G, I, H = 25 µm.

**Figure 3.**
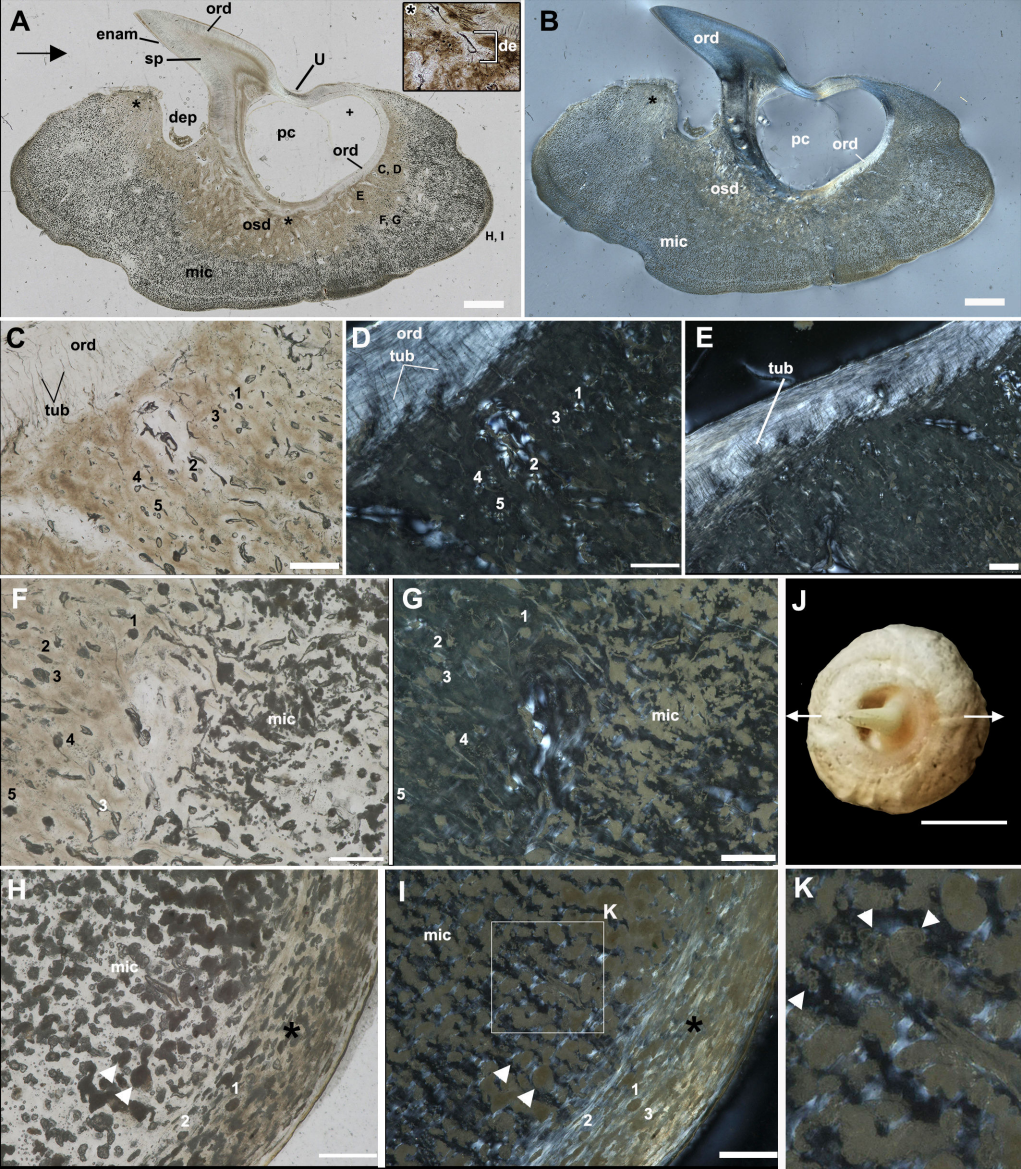
Hard tissue histology of the basal tissues in a buckler of *Raja clavata* (Rajidae, Chondrichthyes, unregistered specimen, NHM Life Sciences collection). A–G, H–K, longitudinal sections in A, C, F, H, white light; B, D, E, G, I, K, cross-polarized light. J, Image of sectioned buckler in dorsal view. A, B, Similar to buckler in figure 5, but with a thicker region of osteodentine (denteon shown in inset A from the osteodentine) and multiple layers of orthodentine in the spine. Asterisk indicates the region where the close-up image of denteons in the osteodentine layer was taken; in A, letters C–I indicate regions shown in close-up in the rest of the figure. C, D, Spine base (orthodentine) and osteodentine, in cross-polarized light; (D) denteons in the osteodentine surrounded by fibres organized in a Maltese cross pattern; this is less apparent further along the spine base (E); F, G, transition from the osteodentine to the microcancellous tissue, fibre arrangement is lacking in cross-polarized light (G); H, I, K, base of the buckler with a distinct outer layer, rich in intrinsic fibres that parallel the buckler base (asterisk). Small spaces are present within this fibre layer (1–3), surrounded by the fibres. White arrowheads indicate large spaces with the microcancellous tissue (H, I), with potential evidence of tissue resorption (K, white arrowheads). de, denteon; dep, depression; enam, enameloid; mic, microcancellous tissue; ord, orthodentine; osd, osteodentine; pc, pulp cavity; sh.f, Sharpey’s fibres; tub, dentine tubules; numbers 1−5 indicate equivalent spaces within the osteodentine (C, D, F, G) and in the basal tissue (H, I). Scale bars: A = 1 mm; C–I = 100 µm; J, K = 0.5 cm.

**Figure 4.**
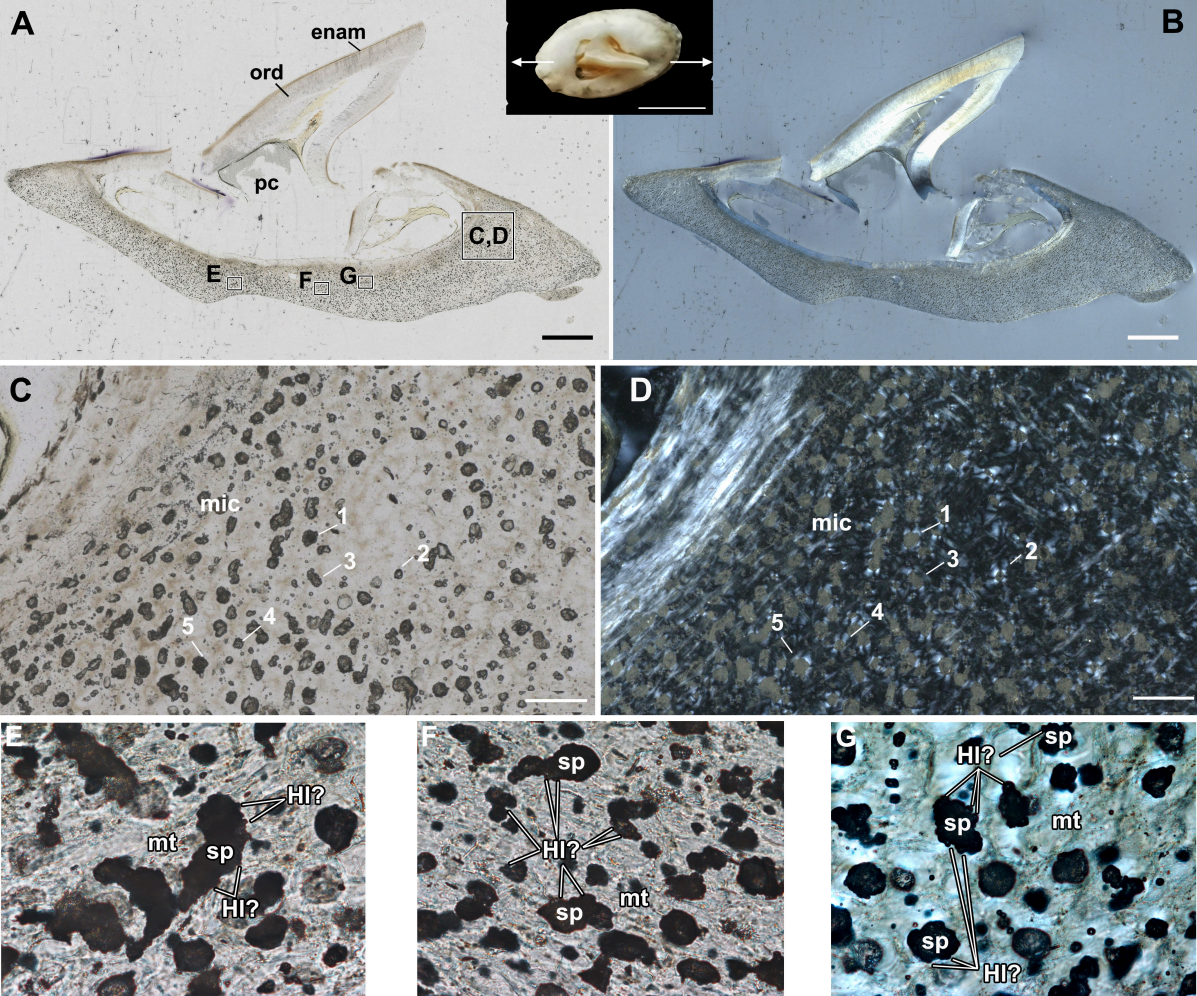
Small buckler of *Raja clavata* (Rajidae, Chondrichthyes, unregistered specimen, NHM Life Sciences collection) with a less expanded base. A, Longitudinal section under plane-polarized light of the buckler shown in dorsal view in the inset. B, Longitudinal section in cross-polarized light. C, D, Region of microcancellous tissue with rounded spaces and showing a Maltese cross arrangement of fibres around the spaces (D). E, Close-up of microcancellous tissue showing spaces with irregular walls, reminiscent of Howship’s lacunae. F, Close-up of a second region with putative Howship’s lacunae around the enlarged spaces. G, Cross-polarized light close-up image of microcancellous tissue showing putative Howship’s lacunae. enam, enameloid; Hl?, putative Howship’s lacunae; mic, microcancellous tissue; mt, matrix; ord, orthodentine; pc, pulp cavity; sp, spaces in microcancellous tissue; numbers 1−5 indicate equivalent spaces within the basal tissue (C, D). Scale bars: A, B = 1 mm; C, D = 100 µm.

**Figure 5.**
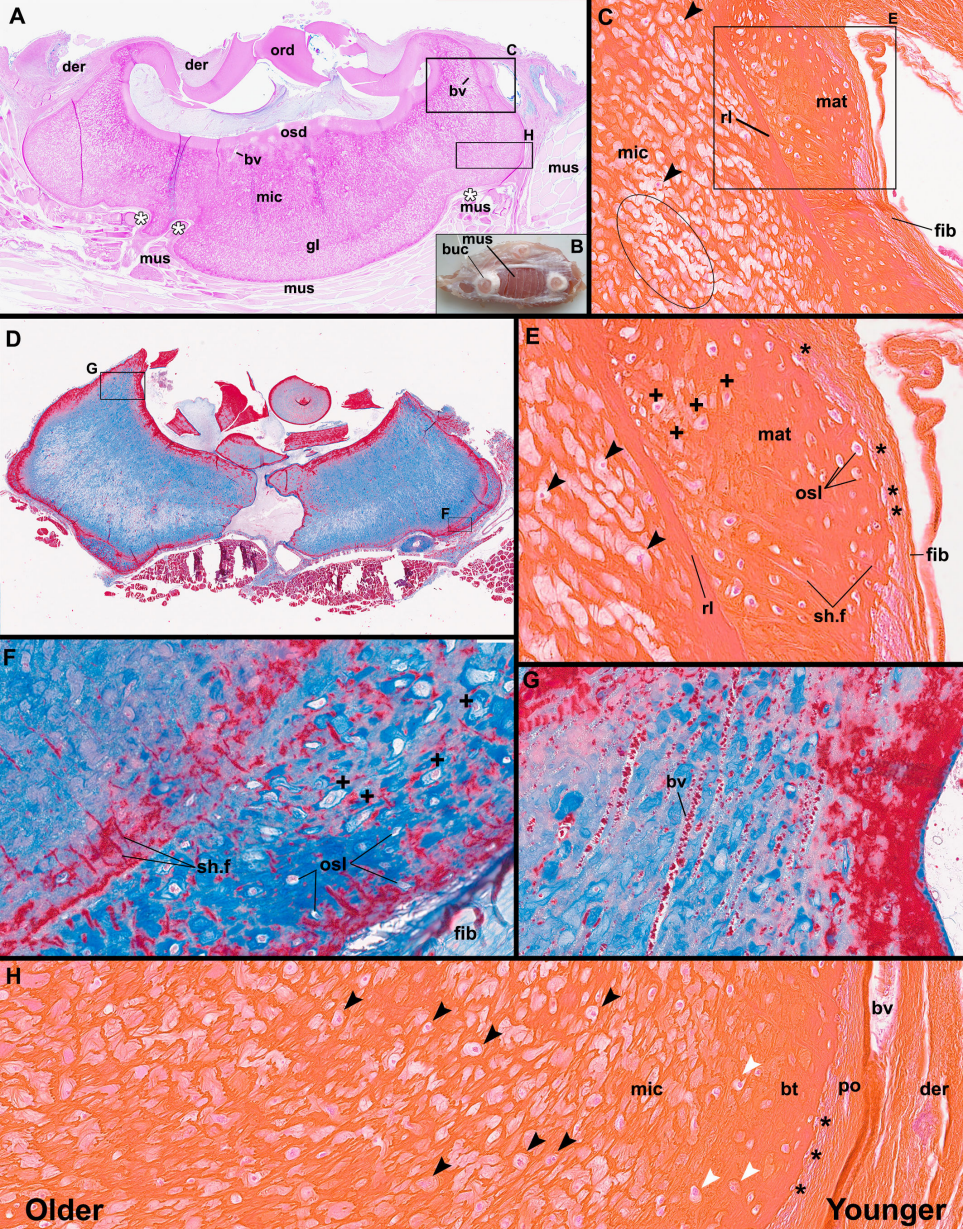
Paraffin histology of bucklers from a *Raja clavata* (Rajidae, Chondrichthyes), France. A, C, D–F, Decalcified, stained longitudinal sections; B, macrophotograph of *Raja* pectoral fin with *in situ* bucklers. A, Buckler section with broken spine (PAS-BA stain), white asterisks indicate regions of buckler base growing over the fin musculature; C, dorsal region of buckler (Hematoxylin & Eosin stain) showing three distinct regions (mic, mat, fib) indicating different stages of buckler development. Black arrowheads indicate single cells within the microcancellous spaces. Circled region shows a cell-free region of large spaces and sparse matrix; D, second stained buckler (Masson’s trichrome) section; E, close-up of the interface between the three major regions of the buckler base in C. Black asterisk indicates matrix-producing cells being engulfed by matrix (along with thicker fibres, entombed cells are aligned between these fibres). ‘+’ Indicates angular cell spaces, potentially indicating osteocytic osteolysis; F, close-up of ventral surface of buckler in D showing regions of peripheral bone-like layer with osteocytes and more internal layers of maturing microcancellous tissue with enlarged cell lacunae, suggestive of osteocytic osteolysis (+); G, section comparing blood vessel spaces in the buckler to the microcancellous spaces; H, transect through the buckler base in A (but with H&E stain), showing histological interpretations of the major tissue types forming the base. Black arrowheads identify cells within the microcancellous tissue. White arrowheads mark potential transitional cells with enlarged lacunae, interpreted as mononucleated cells. bt, bone-like tissue; bv, blood vessel; der, dermis; fib, fibrous layer; mat, mineralized matrix; mic, microcancellous tissue; ord, orthodentine; osd, osteodentine; osl, osteocyte lacunae; po, periosteum-like layer; rl, rest line; sh.f, Sharpey’s fibres; sp, spaces in microcancellous tissue.

**Figure 6.**
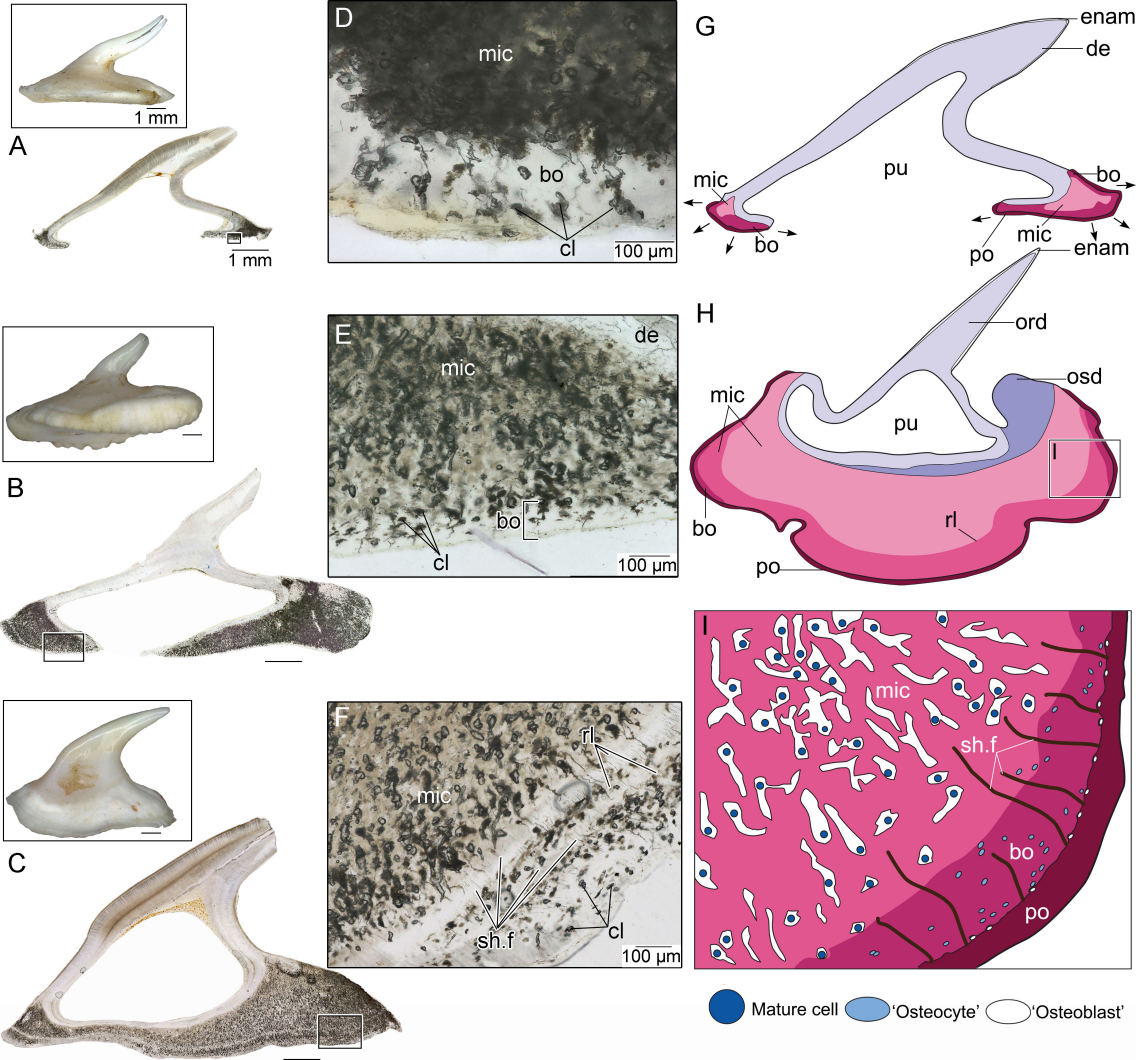
Hypothetical growth series of bucklers and thorns, schematic illustration of young and mature bucklers, highlighting the growth of the basal tissues. A, Small scale (buckler or thorn) with incipiently developed basal tissue and an open pulp cavity in longitudinal section. B, Slightly larger scale with more extensively developed basal tissues and a partially enclosed pulp. C, Larger scale with a nearly completely enclosed pulp and thicker mass of basal tissue. D, Close-up of periphery of basal tissue in A, showing outer bone-like and inner microcancellous tissues. E, Close-up of periphery of basal tissue in B, showing outer bone-like and inner microcancellous tissues. F, Close-up of periphery of basal tissue in C, showing outer bone-like and inner microcancellous tissues separated by multiple rest lines. G, Illustration of a young scale with interpretation of basal tissues and their growth directions. H, Overview illustration of a mature buckler. The basal tissue is formed of successive layers of mature, microcancellous tissue (light pink), which is derived from the same cellular bone-like tissue forming along the periphery (dark pink) in the youngest scales (G). I, Illustration of the formation and maturation of the basal buckler tissues, highlighting the formation of the cellular bone-like tissue from the periosteum-like layer (red) and the distribution of the three cell types (‘osteoblast’, ‘osteocyte’ and mature cells). bo, cellular bone-like layer; cl, cell lacunae; de, dentine; enam, enameloid; gl, growth line; mic, microcancellous tissue; ord, orthodentine; osd, osteodentine; po, periosteum-like layer; pu, pulp; sh.f, Sharpey’s fibres.

Bucklers from the extant thornback ray (also referred to as the thornback skate), *R. clavata*, include a single isolated specimen that was CT scanned and sectioned (Life Sciences collection, Natural History Museum, BMNH; figures 1 and 2), and a partial specimen that was CT scanned for quantification of the spaces within the basal tissue (electronic supplementary material, figure S1). Seven additional bucklers and thorns were sectioned for hard tissue histology (four from the unregistered samples in the Life Sciences collection, BMNH; figures 2–4, electronic supplementary material, figures S2 and S3; three unregistered specimens from the C. Underwood collection, Birkbeck, University of London, UK; figure 6). In addition, two female specimens were investigated with respect to thorn and buckler distribution on the body (BMNH 85.11.3.4; BMNH 64.4.26:87-8; figure 1A,B), and an additional live individual was caught and released by C. Underwood after photographing buckler distribution (figure 1C). To investigate bucklers *in situ*, pectoral fins with intact bucklers for histological study were purchased at a fish counter in France (the exact geographical origin of these individuals is therefore unknown; figure 5).

### (b) CT scanning

A single buckler specimen was CT scanned in Science Innovation Platforms, Natural History Museum (NHM), on a Zeiss Radia Versa equipped with a 10 W sealed transmission source at 100 kV, 10 µA and a HE1 source filter. 2023 projections were generated at 7.1 µm voxel size. Scans were rendered using Avizo 3D 2021.2 (https://www.thermofisher.com/uk/).

To isolate and quantify spaces within the microcancellous tissue, we performed a µCT scan of a fractured air-dried buckler with an EasyTom scanner (RX Solutions, Chavanod, France) at 100 kV source voltage and 83 μA source current, with 1440 projection images taken per turn at 2 frames per second and 15 frames averaged per projection image. The scan focused on an approximately cubic volume of interest (VOI, ~1 mm^3^) deep to the pulp cavity (electronic supplementary material, figure S1A–C); reconstructions were performed with RX Solutions software with beam hardening correction, resulting in an isometric voxel size of 4.64 μm. Visual data were processed in Amira ZIB Edition software to identify voids. We first applied the *Local Normalization* module to uniformize the mean and variance of the greyscale data, then selected (labelled) only the mineralized tissue, excluding internal voids, using the *Interactive Thresholding* module (electronic supplementary material, figure S1D). An *Ambient Occlusion* step was then applied to this selection to identify those background voxels largely surrounded by foreground voxels (mineral); a thresholding applied to the resultant ambient occlusion field allowed selection of all internal voids—both buckler voids and vascular channels—including those at the edges of the VOI and therefore open and connected to the background. The *Connected Components* module was then applied to the segmented voids to divide that binary label field into individual, non-conjoined spaces; however, most voids remained connected as a single mass (electronic supplementary material, figure S1E), due to the very thin mineralized septa between adjacent spaces making them appear linked at our scan’s resolution. A *Contour Tree Segmentation*, run on a random walk distance map of the label field resulting from the previous step, allowed final isolation of individual voids (electronic supplementary material, figure S1F). *The Filter by Spreadsheet* module was used to further filter irrelevant morphologies based on their size, removing especially large objects (e.g. vascular channels) and small ones (e.g. partial labels, noise). A manual proofreading step verified that the remaining objects (electronic supplementary material, figure S1C,G) were the internal voids we intended to capture, of similar size and shape as those observed in histological data. Finally, a *Label Analysis* module applied to the segmented labels provided morphometrics of all remaining individual objects/voids (linear dimensions, volume; electronic supplementary material, figure S1H), as well as the orientations of their long axes relative to the direction of the buckler’s spine (based on their absolute eigenvector in the scan’s *Y* direction; electronic supplementary material, figure S1I).

### (c) Ground sectioning

Isolated bucklers and thorns (dry) in figures 2–4 and electronic supplementary material, figures S2 and S3, were sectioned in Science Innovation Platforms, NHM. Bucklers were mounted in Kemet KEPT resin and left to cure in a pressure chamber for 24 h. The cured resin block was then levelled using a Petro-Thin grinding machine. The buckler was then exposed from the resin by grinding using a twin-plated lapping machine using progressively finer grits of abrasive silicon carbide papers. The exposed and smoothed buckler surface was then mounted onto a glass slide using Loctite AA358 UV resin and cured for 10 min using a UV light source. The mounted sample was then cut and ground down to an approximate thickness of 250 µm using a Geo-form sectioning machine. The section was then lapped by hand to an approximate thickness of 60 µm using P2500 and P4000 silicon carbide powder. A coverslip was then applied using Loctite AA358 UV resin. Bucklers and thorns in figure 6 were sectioned at King’s College London. Samples were embedded in Epothin 2 epoxy resin and placed under vacuum. Cured resin blocks were then sectioned using a Buehler Isomet 1000 slow-speed saw, ground using P2000 grit silicon carbide grinding foils and mounted onto acrylic slides using Scotchweld SF100 cyanoacrylate glue. Mounted samples were then cut using the Isomet 1000 to a thickness of 700 µm and hand-ground down to the desired visual clarity using P1200, P2000 and P4000 silicon carbide foils.

### (d) Paraffin sectioning and staining fresh samples

Two bucklers from a fish market in France were sampled with surrounding muscle tissue and fixed in 4% paraformaldehyde in phosphate buffer saline 1× (PBS 1×) for 48 h, quickly rinsed in PBS 1× and decalcified for 48 h in Epredia™ ShandonTM TBD-2TM. The whole bucklers were progressively dehydrated in graded PBS 1×/ethanol baths (50%, 75% and three times 100%) before inclusion in paraffin. The 7 µm serial paraffin sections were placed on three separate slides to allow for three standard histological stainings:

Haematoxylin Eosin Saffranin (HES): cell nuclei are purple; cytoplasm is light pink, thick collagen fibres (e.g. Type I) are bright orange/red ‘collagen fibres’.

Masson’s Trichrome (TM): collagen-based extracellular matrices are blue, fibrous/dense cytoplasm is red (e.g. muscle fibres, keratinocytes, red blood cells).

Periodic Acid Schiff-Blue Alcian (PAS-BA): acid glycosaminoglycan-rich matrices stain deep blue (e.g. cartilaginous hyaline matrices and mucus cells), any polysaccharides stain pink to fuchsia.

Histological stained sections were imaged at 40× on a slide scanner (Nanozoomer 2 Hamamatsu).

### (e) Energy dispersive spectroscopy

The sagittally cut section from figure 3 was coated with a thin layer of carbon and energy dispersive spectroscopy (EDS) was carried out by an electron probe micro-analysis microscope (Zeiss Evo 15LS) in Science Innovation Platforms, NHM. Distribution mappings for calcium (Ca), phosphorus (P) and magnesium (Mg) were analysed under an operating voltage of 20 Kv and probe current of 0.44 nA (electronic supplementary material, figure S2).

### (f) Digital microscopy

Ground sections of extant *Raja* bucklers and thorns were imaged in the Faculty of Dentistry, Oral & Craniofacial Sciences at King’s College London using a Keyence VHX-7000 digital microscope. Sections were imaged under varying combinations of plane- and cross-polarized transmitted light, as well as plane- and cross-polarized incident coaxial light, depending on the thickness of the sections and the desired magnification.

## 3. Results

### (a) Distribution and gross morphology of the bucklers in *Raja clavata*

Adult and subadult female *R. clavata* have numerous bucklers on the dorsal (figure 1A,B) and ventral surfaces of the body (figure 1C), although these were absent in juvenile specimens studied (C. Underwood, personal observation, 2024). Although BMNH 64.4.26:87-8 falls into the size range given for sexually mature *R. clavata*, BMNH 85.11.3.4 does not (720−930 mm total length (TL); [17]). The individual in figure 1A is smaller, possesses different coloration patterns, and is presumed to be younger than the individual in figure 1B (BMNH 85.11.3.4; 450 mm TL; BMNH 64.4.26:87-8; approximately 900 mm TL). In this younger specimen, the bucklers are positioned at specific sites, and paired medially and symmetrically along the body. However, they are absent from around the orbits, do not form rows, and are present on the terminal tail. Thorns (lacking the expanded buckler base) are uncommon, except for a few along the body (figure 1A). In the putatively older specimen, the bucklers are more numerous and a single pair of bucklers occurs near the spiracular opening (bu.sp, figure 1B). In the larger specimen, many more thorns are present, particularly along the body and at the edges of the fins. The basal surface of all bucklers is strongly convex and embeds deep within the tissue of the torso and pectoral fins.

Excised bucklers have the appearance of a recumbent denticle with a massive, bulbous collar, typically chalky white in appearance. A single CT scan of a buckler approximately 1.5 cm long (measured across the greatest length of the buckler, through the spine) illustrates the very expanded base that dorsally encroaches upon the spine (figure 1D), representing a periodic expansion, as shown by radiopaque increments, consistent with rest lines, preserved inside the buckler base and on the surface (figure 1E−H). This expansion alters the shape of the pulp cavity of the spine, forming a distinct ‘U’-shaped furrow anteriorly and a deep pit below the now more posteriorly aligned spine (figure 1E); the buckler in dorsal and lateral view (figure 1D,F) illustrates how the spine appears sunken into the base. Virtual sections of these CT scans show that regions of higher mineralization (brighter) are found in the spine and central part of the base (figure 1E,G,H), the posterior margin of the base (figure 1D–H, asterisk) and in the rest lines of the buckler base (figure 1E–H).

### (b) Energy dispersive spectroscopy

EDS analysis was performed to determine the general composition of the buckler tissues and also to determine if the unusual basal structure of the buckler has a similar elemental composition to other mineralized structures in chondrichthyans, for example, the presence of Mg-rich whitlockite as in the dentition of holocephalan chondrichthyans [18,19]. From distribution maps of Ca, P and Mg in the sagittal section of the buckler (electronic supplementary material, figure S2), it is evident that, although Ca and P are found in all the tissues of the buckler, their concentrations are not homogeneous. Both elements present a higher concentration in the tissues around the pulp cavity (electronic supplementary material, figure S2A,B), as indicated by the yellow/orange colours for Ca (electronic supplementary material, figure S2A) and pink for P (electronic supplementary material, figure S2B). Ca and P exhibit lower concentrations in the basal portion of the buckler, except along the rest lines. The basal tissues contain many voids with no elemental information, suggesting they are empty in dried sections. Mg is also present with a homogeneous distribution in the buckler, but in a lesser concentration than Ca and P (electronic supplementary material, figure S2C, blue).

### (c) Buckler and thorn hard tissue histology

Sagittal sections through isolated bucklers and thorns were examined under white and cross-polarized light (figures 2–4, electronic supplementary material, figure S3). The spine of the bucklers and thorns is composed of classical tubule-rich orthodentine capped with thin layers of enameloid (figures 2C,E–G and 3C,D). Surrounding the orthodentine is a mineralized tissue that contains fibres extending from the outside of the spine base and inserting into the buckler base, interpreted as Sharpey’s fibres (figure 2B). Immediately adjacent to the orthodentine at the base of the spine is a thin layer of osteodentine, characterized by small denteons (figures 2E and 3A); osteodentine also continues to the posterodorsal buckler surface (figure 2A,D).

The characteristically swollen base of the buckler is composed of a thick tissue; a series of indentations posteriorly (figure 2A) represent the surface manifestations of rest lines shown in figure 1. The basal tissue is dominated by irregularly shaped structures that contain no mineral (electronic supplementary material, figure S2) and are therefore empty spaces in dried sections (figure 2F–N). Based on this tissue’s appearance, we refer to this basal layer as ‘microcancellous tissue’ (*sensu* [9]). The spaces within this tissue are often of a large, complex shape, smooth-walled and larger than a typical bone cell lacuna in other vertebrates, with no signs of cell canaliculi. These observations are inconsistent with cell lacunae in bone or denteons in osteodentine. In the upper and middle region of the buckler base (figure 2F–H,K), the spaces are more numerous and densely packed within the tissue and appear to have a preferred orientation towards the pulp cavity of the spine. More ventrally (figure 2I,J–N) the spaces are less densely packed and lack a preferred orientation. Under cross-polarized light (figure 2J,M,N), the pattern of non-birefringence indicates that the intrinsic tissue fibres between the spaces are oriented in different directions (figure 2M,N).

A second buckler demonstrates the same arrangement of tissues (figure 3). The buckler base surrounds the spine, producing the ‘U’-shape of the anterior spine base and depression of the base surface posteriorly (figure 3A,B). The spine is composed of thick layers of orthodentine, and a continuous orthodentine layer lines the pulp cavity ventrally at the base of the spine (figure 3A–E). Below this orthodentine, denteons and osteodentine can be recognized (de, osd, asterisk, figure 3A,B). Under cross-polarized light, the small denteons show a fibre arrangement reminiscent of the Maltese cross pattern (figure 3D), but others do not (figure 3E–G). Below this is the thick microcancellous tissue, containing numerous enlarged spaces (figure 3H,I). None of these tissue spaces show a Maltese cross pattern of fibre arrangement in the surrounding matrix and instead appear to cross-cut the surrounding matrix, forming irregular voids in the surrounding tissue (figure 3H–K). Along the periphery of the buckler base is a thin layer of denser tissue containing smaller spaces that are more reminiscent of cell lacunae (black asterisk, figure 3H,I). Under cross-polarized light, the intrinsic matrix fibres in this outer layer are oriented parallel to the base of the buckler.

A third, smaller buckler shows the same dorsal overgrowth and impingement on the buckler spine, but with a less swollen buckler base (figure 4A,B). The spine is broken, but a thin layer of enameloid is present, coating the orthodentine of the spine. Below the spine is a thinner region of the microcancellous tissue compared with the larger bucklers, with no apparent growth lines. Here, some of the spaces are surrounded by fibres arranged concentrically, forming a Maltese cross pattern under cross-polarized light, and the spaces more frequently retain a more rounded shape (figure 4C,D). However, higher magnification views of some of the spaces in the microcancellous tissue show evidence of scalloping along their peripheries (figure 4E,F). These scallops appear to truncate the surrounding matrix under cross-polarized light and are reminiscent of Howship’s lacunae following resorption or removal of mineralized tissue (figure 4G).

An isolated thorn was also investigated to determine whether this unusual microcancellous tissue is solely found in bucklers in *R. clavata* or is more widespread in their dermal skeleton (electronic supplementary material, figure S3). Despite structural and topological differences with bucklers, thorns possess the same arrangement of tissues, with only minor differences. There is only a slight ‘U’-shaped bend in the spine anteriorly and a small depression posteriorly (electronic supplementary material, figure S3A–C). Microcancellous tissue also forms the base of the thorn as it does in the buckler, and rest lines are similarly present (electronic supplementary material, figure S3D–G). The microcancellous tissue is similarly characterized by an abundance of spaces, some are rounded spaces, whereas others are more irregular, and as above are comparable to Howship’s lacunae in bone (electronic supplementary material, figure S3F,G). Along the periphery of the thorn base and external to a rest line, the matrix type shifts dramatically and the spaces are oriented more circumferentially, more similar to cell lacunae (electronic supplementary material, figure S3D–G).

### (d) Buckler paraffin histology

To determine the developmental and cellular origins of the various buckler tissues, *in situ* bucklers from the pectoral fin of a freshly caught ray were sectioned and stained using HES, TM and PAS-BA (§2). These sections show that each buckler is firmly anchored in the dermis (der, figure 5A), with dermal tissue extending into the depressions formed by the overgrowth of the spine described previously. Fin muscles are present between the bucklers (figure 5A,B), but also underlie the buckler, having different orientations along the base; the shape of the buckler base is closely associated with the shape of the underlying muscle (figure 5A). The spine of the denticle was partially removed when the animal was collected (figure 5B), but shows typical orthodentine and a small area of osteodentine at the base of the spine (figure 5A). The most conspicuous part of the buckler base is the microcancellous tissue, which contains several entombed blood vessels and one apparent rest line (figure 5A,G). Unlike the spaces in the microcancellous tissue, the vascular channels appear more elongate, smooth-walled and approximately the same diameter (figure 5G). Along the periphery of the base, this rest line separates denser layers of tissue more externally from the main microcancellous tissue of the buckler base (figure 5A–E). A band of fibrous tissue extends along the edge of the buckler (figure 5C,E), covering the entire buckler base. Most of the fibres are parallel to the buckler base; however, larger fibre bundles curve towards the buckler and are anchored to the mineralizing buckler base (figure 5E). The mineralized ends of these fibre bundles within the buckler base penetrate deeply into the microcancellous tissue, consistent with Sharpey’s fibres (figure 5E,F). At the interface between the fibrous outer layer and the buckler base, cells are either partially or completely entombed within the mineralizing matrix in the newly formed layer of buckler tissue. Nearly all the spaces within the outermost layer of the buckler base contain cells. In this region, we therefore refer to the spaces in the tissue as true cell lacunae.

The rest line separates this peripheral cell-rich region of the growing buckler from the inner microcancellous tissue, which forms the bulk of the buckler base (figure 5E). In the outer regions, matrix-depositing cells are apparently entrapped within the centrifugally deposited matrix (figure 5C,E). Sharpey’s fibres originating from the fibrous outer covering extend through the mineralized buckler base, suggesting that the centrifugally deposited matrix also incorporates these fibre bundles into the matrix. The cells are aligned in clusters between the Sharpey’s fibres (figure 5E).

The nature of the lacunae also changes during the maturation of the basal buckler tissue, internal to the peripheral cell-rich matrix region; in figure 5E,F, the ‘+’ signs mark points where lacunae have become irregularly shaped and angular, with some of the enlarged spaces still occupied by cells. In other cases, the cells appear to be preferentially occupying one side of their larger lacunae (figure 5H, white arrowheads).

A second transition occurs in a more internal region (figure 5C,E, internal to the rest line). In this region, the matrix is perforated by numerous large spaces, only some of which contain cells (black arrowheads, figure 5E,H). The spaces differ from the elongate vascular channels that extend through the buckler base (figure 5G) and are oriented in the same direction as the Sharpey’s fibres from the outer regions of the bone-like buckler tissue, and the cells between these. However, the spaces in the more internal regions are larger and more irregular in shape, with rounder walls. In some areas, the spaces appear to have joined, forming large regions of cell-poor, microcancellous tissue (figure 5C). The spaces in the interior of the buckler base also stain more weakly in all three stains, presumably due to the paucity of matrix and entombed cells.

In other regions of the buckler, the transition between younger cell-rich tissue and the older microcancellous region is more gradual (figure 5F,H). Here, the difference in cell lacuna and space size is still clear. The entrapped cells of the peripheral tissue are much smaller than those found in the more sparsely populated microcancellous tissue. Moreover, the distances between neighbouring cells appear to be much larger in the microcancellous tissue compared with the cellular tissue forming along the periphery of the buckler base (figure 5H).

### (e) Orientations and sizes of void spaces in the buckler

Based on the quantitative analysis of a µCT VOI, the buckler base is extremely porous, with voids occupying a high proportion of the tissue (~63.8% by volume). Volume-rendered voids were lobulated, with three-dimensional morphologies appearing similar to dripping beads of candle wax (electronic supplementary material, figure S1F,I inset). The exceptionally high density of voids and the narrowness of intervening mineralized septa made it challenging to verify whether some labelled objects in our final segmentation were isolated elements or in fact several cavities that had been erroneously selected/merged together. To provide more accurate measurements of individual void dimensions, we therefore further filtered our data to include only the more linear cavities (i.e. elongate, with similar width and depth; electronic supplementary material, figure S1I), thereby excluding aggregate labels potentially representing multiple voids. The ‘linear voids’ were 167.9 ± 29.5 µm long, with diameters of 99.4 ± 17.1 µm × 72.9 ± 11.8 µm (figure S1H); the latter measurements are in keeping with our histological data (figure 5), where cells of similar size were observed to typically fill the diameter of the voids they occupied. Finally, analysis of the alignment of the long axes of the ‘linear voids’ showed a dominant orientation towards where the buckler’s spine would be (i.e. the scan’s *Y*-axis; electronic supplementary material, figure S1I), indicating a preferred orientation and structural anisotropy to the porosity in the VOI’s region of the buckler.

### (f) Growth and maturation of the buckler and thorn bases

Given the unusual nature of the tissues of the buckler base, we then made ground sections of three enlarged scales (either bucklers or thorns at early stages of formation, which are indistinguishable from each other) with varying degrees of basal tissue formation to understand the relationship between the microcancellous and cell-rich peripheral layers through ontogeny (figure 6A–F). The smallest scale had only a fringe of basal tissue surrounding the spine, with a large ventral opening for the pulp (figure 5A, electronic supplementary material, figure S4A–C). Thin sections revealed that most of the tissue was consistent with the microcancellous tissue we characterized for mature bucklers and thorns. However, the outermost layers contained only small spaces, likely cell lacunae (figure 6A,D). The second scale possessed a more enlarged base with a single rest line along its outer fringes (electronic supplementary material, figure S4D–F). The pulp cavity remained open ventrally albeit to a lesser extent due to basal tissue growth. In the thin section, the basal tissue again consisted mostly of the microcancellous tissue, but with an outermost fringe of tissue with small lacunae (figure 6B,E). The third scale had a nearly enclosed pulp with a basal tissue containing multiple rest lines visible along the surface (electronic supplementary material, figure S4G–I). In the thin section, the base consisted almost entirely of microcancellous tissue, but with a clear outermost layer of denser tissue with cell lacunae. Sharpey’s fibres were also visible in multiple layers of the bone-like tissue (figure 6C,F).

Combining these data with our hard tissue and paraffin histological observations demonstrates that the basal tissues grow centrifugally away from the central spine of the bucklers and thorns (figure 6G). Moreover, the peripheral layers, which are the newest layers to form, are very similar to cellular bone with large numbers of entombed cells and Sharpey’s fibres from the surrounding fibrous tissue along the mineralization front. At no point did we find the microcancellous tissue along the periphery of the scales. We also did not find bone-like tissue in deeper regions of the bucklers and thorns. Therefore, after the formation of an initial bone-like layer, it appears it is somehow remodelled into the mature microcancellous tissue that comprises most of the buckler and thorn bases (figure 5G–I).

## 4. Discussion

Reif [9] identified acellular and microcancellous types of bone along the bases of the bucklers in the extant batoid, *Raja*, suggesting that at least some chondrichthyans are able to produce a form of bone in the odontode elements of the dermal skeleton. Our reinvestigation of the bucklers of extant *R. clavata*, combining hard tissue histology with paraffin sections of fresh material indicates that the peripheral tissue is remarkably similar to cellular bone [20, figs. 6–30]. We believe Reif’s [9] conclusion stemmed from interpretations of hard tissue sections where the cellular component would not have been visible. Our paraffin sections instead demonstrate that *R. clavata* does indeed form a cellular tissue along their buckler bases that is consistent with bone. Our examination of scales at different stages of ontogeny also indicates that the bone-like tissue is subsequently modified into a novel mineralized tissue for which we retain Reif’s [9] term ‘microcancellous tissue’. Furthermore, through fresh and fixed tissue analysis, we can infer changes in cellular activity within the buckler base by observing cellular organization and the surrounding matrix in stained thin sections.

Given that the dermal skeleton would not contain any cartilaginous precursor during scale development (unlike in the vertebrae of some elasmobranchs [12]), the outer fibrous membrane surrounding the bucklers and thorns is more appropriately termed a ‘periosteum-like’ tissue. Moreover, the peripheral cells form an extracellular matrix that eventually envelops each cell and some of the surrounding vasculature, leading to the formation of cellular, vascularized tissue that initially resembles intramembranous bone (figure 5E,H). Some of the Sharpey’s fibres within the base originate from large collagen fibre bundles that anchor the bucklers into the surrounding fibrous tissue (‘dermis’ as identified by Reif [9]); however, many of these Sharpey’s fibres are actually attachment sites for the periosteum-like tissue surrounding the base, similar to intramembranous bone (figure 5E) [20]. The Sharpey’s fibres also restrict the entombed cells into centrifugally oriented clusters, similar to the columnar spaces seen in the more mature microcancellous tissue.

Whereas the initial deposition of the basal tissue of the bucklers and thorns resembles cellular bone, the maturation of this into the microcancellous tissue in the deeper regions of the bases is puzzling. The buckler and thorn bases consist mainly of this microcancellous tissue, which must be derived from the more recently formed bone-like material (figure 5). However, the morphology of the spaces is peculiar; the resident cells occupy spaces that are larger and more elongate than the cell lacunae of the outer bone-like layer. These spaces are often many times larger than the cell bodies (figure 5E,H), suggesting that the surrounding matrix has been modified through cellular activity, leading to the formation of oversized, irregular-shaped spaces in which the cells reside. Some of these larger spaces truncate the surrounding matrix (figure 2M,N) and, in some cases, have irregular, scalloped walls (figures 3H–K and 4E–G). These features are reminiscent of bone resorption, but we found no evidence for bone resorption by multinucleated osteoclasts [21]. One explanation that is more consistent with the histology of the buckler tissues is osteocytic osteolysis. This process involves the localized and selective resorption of bone, but by osteocytes rather than osteoclasts, and may play a significant role in mineral metabolism similar to that in other vertebrates with cellular bone: this process has been documented in mammals [22], reptiles [23,24], bony fish [25] and early vertebrates [26].

Buckler base cells may therefore transition from matrix-producing cells to matrix-remodelling cells (e.g. the cells marked by white arrowheads, figures 5H and 6I). Each space in the microcancellous tissue is mostly empty in section and only occasional cells are visible across the entire base of a buckler (figure 5H). These differentiated cells may therefore somehow move within the buckler base tissue over time, creating elongate, smooth-walled spaces containing individual cells. Confirming the resorptive behaviour of these cells would require tartrate-resistant acid phosphatase (TRAP) staining of fresh, fixed tissues to see if differentiated cells within the buckler base are able to demineralize the surrounding matrix; however, our sample preparation of soft tissue specimens did not permit the use of this stain. The functional significance of this differentiation of cells within the base of each buckler is, at present, unclear. Regardless, we propose that the presence of matrix-depositing cells developing in the periosteum-like layer of the buckler base, along with the arrangement of fibres around the tissue spaces in the peripheral layers of the buckler support this as a cellular, bone-like tissue (figure 6). At some point, these cells may transition to a state of activity that compares to osteocytes (reduced number of cells enclosed in their mineralized matrix) and may also display some capacity to remodel the matrix. Moreover, the association of matrix-depositing cells and fibres along the periphery of the buckler may be indicative of some type of attachment tissue, otherwise described only for teeth [27]. However, the homology of the large amount of microcancellous tissue beneath the spine is difficult to assess relative to other vertebrates, given the uniqueness of this tissue.

It is well-established that fossil relatives of chondrichthyans had bone (the acanthodians or ‘spiny sharks’ [28,29]) and that chondrichthyans, generally lacking bone, may have lost many of the genes involved in bone formation (as, for example, demonstrated in holocephalans) [11,30,31]. However, mineralized tissues have been identified previously, for example, in mineralized tiles (tesserae) covering the cartilaginous skeleton [32–37] and in the neural arches [12,15,36,38,39], some of which have been characterized as bone-like. Indeed, some of the mineralized cellular layers we observed along the peripheries of buckler and thorn bases is very similar to bone-like tissues identified along the centra of extant sharks. However, in these modified scales, there would be no association with cartilage, cartilaginous precursors or tesserae at any point during development. Furthermore, despite acellular bone being identified in the base of chondrichthyan denticles and teeth, these have shown no immune reaction to antigens for type-I collagen, and the collagen fibres were not tightly bundled as seen in bone [39–43]. The modified denticles of *R. clavata* (thorns and bucklers) represent yet another form of mineralization; the initial bone-like matrix and subsequently modified microcancellous tissue suggest cell activities similar to osteoblasts, osteocytes and mineralized matrix remodelling of some form. It is possible that more of the genetic toolbox for bone is retained in chondrichthyans than previously thought, but is only expressed in specific odontogenic regions of the skeleton.

## Data Availability

The CT scan files used in figure 1 are available via DRYAD at the following link: [44] scan files used in electronic supplementary material figure S1 are available at [45]. Supplementary material is available online [46].
